# Microwave absorption properties of pyrolytic carbon nanofilm

**DOI:** 10.1186/1556-276X-8-60

**Published:** 2013-02-07

**Authors:** Polina P Kuzhir, Alesya G Paddubskaya, Sergey A Maksimenko, Tommi Kaplas, Yuri Svirko

**Affiliations:** 1 Research Institute for Nuclear Problem, Belarus State University, 11 Bobruiskaya St., Minsk, 220030, Belarus; 2 Department of Physics and Mathematics, University of Eastern Finland, P.O. Box 111, Joensuu, FI-80101, Finland

**Keywords:** Pyrolytic carbon, Nanofilm, Microwave, Skin depth, Electromagnetic interference shielding, 78.67.Sc, 81.16.Dn, 78.30.-j.

## Abstract

We analyzed the electromagnetic (EM) shielding effectiveness in the Ka band (26 to 37 GHz) of highly amorphous nanometrically thin pyrolytic carbon (PyC) films with lateral dimensions of 7.2 × 3.4 mm^2^, which consists of randomly oriented and intertwined graphene flakes with a typical size of a few nanometers. We discovered that the manufactured PyC films, whose thickness is thousand times less than the skin depth of conventional metals, provide a reasonably high EM attenuation. The latter is caused by absorption losses that can be as high as 38% to 20% in the microwave frequency range. Being semi-transparent in visible and infrared spectral ranges and highly conductive at room temperature, PyC films emerge as a promising material for manufacturing ultrathin microwave (e.g., *K*_a_ band) filters and shields.

## Background

Due to the importance of satellite communication, electromagnetic compatibility in the *K*_a_ band (26 to 37 GHz) has recently become an important concern. The band overcrowding requires enhancing electromagnetic interference (EMI) shielding effectiveness (SE), i.e., development of novel coatings, shields, and filters that prevent degradation of the performance of the systems operating in densely populated EM environment [1,2]. It is worth noting that compared to conventional metal-based EMI shielding materials, using carbon-based conducting composites is advantageous for satellite applications because of their low weight, small thickness, and flexibility [3,4]. These include polymer composites containing exfoliated graphite, graphene nanoplatelets, carbon black, carbon fibers and nanofibers, carbon nanotubes (CNT), and carbon onions. Shielding effectiveness of these carbon-based coating has been extensively investigated in the last decade (see reviews [3,4] and the references therein).

The EMI shielding effectiveness of a material is defined as SE (dB) = 10 log (*P*_t_/*P*_i_) [5], where *P*_t_ and *P*_i_ are the transmitted and incident electromagnetic powers, respectively. Thus, the magnitude of the SE is determined by the material transmittivity, which depends on the absorption, reflection, and scattering losses of the EM energy. In homogeneous materials, absorption and reflection losses dominate the SE. The absorption-related losses in conventional metals are determined by the relationship between the metal thickness and the skin depth, which decreases with the frequency [6]. The reflection occurs due to the impedance discontinuity at metal-air interface. The reflection losses decrease at higher frequencies since material impedance increases. The absorption mechanism predominates when the coating thickness is comparable with the skin depth or at sufficiently high frequencies when the conductivity decreases [6]. Thus, conventional metallic coating being much thinner than EM skin depth should, strictly speaking, be transparent to microwave radiation.

Breakthrough in the EMI technology has been recently made by Bosman et al. [7]. Using a simple equivalent transmission line model for the thin film as a lumped resistor they demonstrated that an ultrathin film may absorb up to 50% of the incident power despite the fact that its thickness is only a small fraction of the skin depth [7].

Very recently, we have demonstrated [8] that the pyrolytic carbon (PyC) films with thickness of several tens of nanometers satisfy the requirements imposed by the theory [7]. Specifically, the PyC film thickness is much smaller than the skin depth, which is much smaller than the wave length. Thus these films should allow one to achieve high SE. We showed in [8] that sheet resistance of these nanometrically thin films is close to that of multilayer graphene flakes [9,10] and carbon nanotubes [11], which have already displayed unique EMI shielding ability [3,4,11,12]. However, in contrast to graphene and carbon nanotubes, a catalyst-free synthesis allows one to deposit PyC films directly on both dielectric and metallic substrates of arbitrary size and shape opening a new route towards fabrication of ultrathin EMI protective coatings with enhanced shielding effectiveness.

In this paper, we study experimentally the EMI shielding ability of an ultrathin PyC film in *K*_a_ band (26 to 37 GHz). The thickness of the film is 25 nm, which is close to the PyC skin depth at 800 nm [13]. We demonstrate that despite the fact that the film is several thousand times thinner than the skin depth of conventional metals (aluminum, copper) in this frequency range, it can absorb up to 38% of the incident radiation.

The paper is organized as follows: the details of sample preparation and microwave (MW) measurements are given in the ‘Methods.’ Experimental data together with their physical interpretation are collected in the ‘Results and discussion.’ The ‘Conclusion’ summarizes the main results as well as some important possible applications of the functional properties of PyC films.

## Methods

### PyC film fabrication

Pyrolytic carbon is amorphous material consisting of disordered and intertwined graphite flakes [14]. The historical and literature review of PyC film production via chemical vapor deposition (CVD) method together with fundamentals of model-based analysis of PyC deposition can be found in [14].

In our experiment, the PyC film was deposited on 0.5-mm-thick silica substrates in a single-step CVD process. The CVD setup consists of a quartz vacuum chamber that was heated by tube oven (Carbolite CTF 12/75/700), and a computerized supply system enabling a precise control of the gas pressure and composition. We employed CVD process with no continuous gas flow inside the chamber to reduce gas consumption and, more importantly, to allow more time for polyaromatic structure formation. The loading of the clean quartz substrate into the CVD chamber was followed by purge filling of the chamber with nitrogen (twice) and then with hydrogen to ensure a clean process. After that the chamber was filled with hydrogen up to the pressure of 5.5 mBar and was heated up to the temperature of 700°C at the rate of 10°C/min. At 700°C, the chamber was pumped down, and the hydrogen-methane gas mixture was injected and heated up to a temperature of 1,100°C. CH_4_/H_2_ gas mixture was kept at this temperature for 5 min and then was cooled down to 700°C. After that the chamber was pumped down, filled with hydrogen at the pressure of 10 mBar, and cooled down to room temperature.

The thickness of the deposited carbon film measured by a stylus profiler (Dektak 150, Veeco Instruments, Tucson, AZ, USA) was as small as 25 ± 1.5 nm. The thickness was averaged over ten different points. Since in our CVD setup there was no gas flow during the graphitization, the CH_4_/H_2_ ratio and pressure change simultaneously affecting the PyC deposition rate [15]. At low pressure, this process was well controllable and enabled deposition of the ultrathin films with prescribed parameters. After the CVD process, both sides of the quartz substrate were covered by the PyC film. In order to characterize the film by microwave measurement, one of the substrate surfaces was cleaned out with harsh oxygen plasma (200 W/20 sccm/3 min). In the present communication, we investigate the electromagnetic properties of PyC produced at 75:20 CH_4_/H_2_ ratio, which corresponds to 25-nm thickness of films.

**Figure 1:**
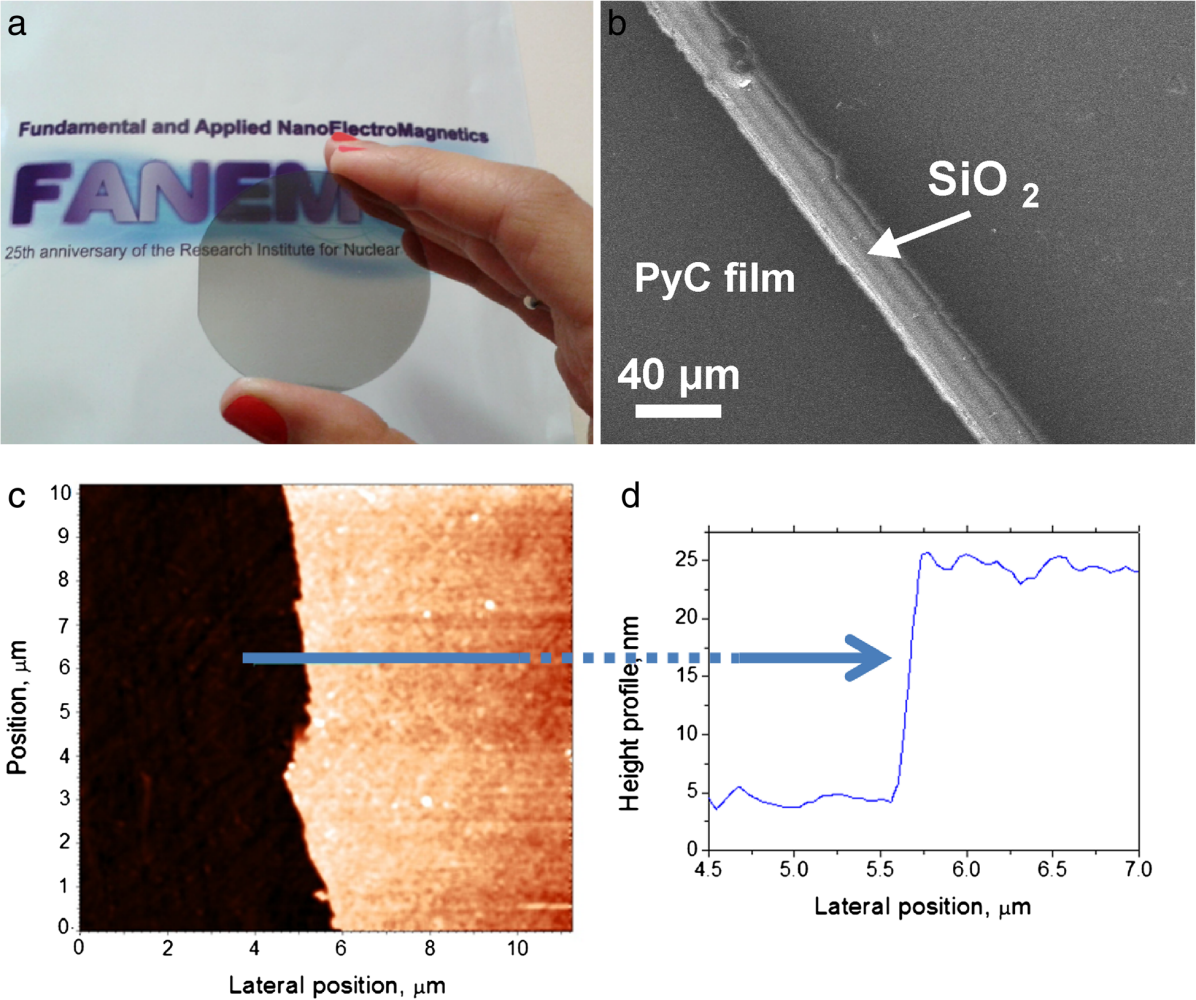
**Optical microscope, SEM, and AFM images. **(**a**) Optical microscope image of PyC thin film of 25-nm thickness deposited on silica substrate. (**b**) SEM image of the film surface area scraped by a blade. AFM image of the PyC film: (**c**) lateral position and (**d**) height profile of the PyC film. Optical image of the PyC deposited film on the quartz substrate is presented in (**a**). Scanning electron microscopy was done by SEM LEO - 1455 Vand and shows good homogeneity of PyC film (**b**). PyC thickness was controlled by AFM (Solver P47 PRO, NT-MDT). Corresponding AFM image of PyC film deposited on the substrate (the lateral position) is presented in (**c**). The height profile (the PyC film thickness) is presented in (**d**).

Optical microscope image of the PyC film deposited on silica substrate is presented in Figure 1a. One can watch that the film is semitransparent. Scanning electron microscopy image of the film was obtained by scanning electron microscopy (SEM) LEO - 1455 Vand (Cambridge, UK). One can observe from Figure 1b that the PyC film shows a good homogeneity. In addition to a stylus profiler data, PyC thickness was controlled by atomic force microscope (AFM; Solver P47 PRO, NT-MDT, Moscow, Russia). The PyC film was scraped by a blade avoiding damage of the SiO_2_ substrate. The AFM image of the PyC film fabricated on quartz substrate (Figure 1c) shows a sharp step-like edge allowing us to perform independent measurement of the film thickness. The lateral position of scratch in the PyC film and the height profile (i.e., PyC film thickness) are presented in Figure 1c,d.

Raman spectroscopy measurements reported elsewhere [8] revealed that morphologically thin PyC film produced at our experiment is composed of randomly intertwined graphite crystallites of the size less than 5 nm but also consisting small amounts of amorphous carbon and *sp*^2^*sp*^3^ bonds [8].

### MW characterization settings

The microwave measurements were made using a scalar network analyzer R2-408R (ELMIKA, Vilnius, Lithuania), including sweep generator, waveguide reflectometer, and indicator unit (personal computer). The IEC 62431:2008(E) standard specifying the measurement method for the reflectivity of EM materials was used. The EM response the PyC fim as ratios of transmitted/input (*S*_21_) and reflected/input (*S*_11_) signals has been measured within 26- to 37-GHz frequency range (*K*_a_ band). The frequency stability of the oscillator was controlled by frequency meter and was as high as 10^−6^. The power stabilization was provided on the level of 7.0 mW ± 10 μW. Measurement range of EM attenuation was from 0 to −40 dB. Basic measurement errors of EM attenuation over the range 0 to 25 dB was *δ*|*S*_21_| = ±(0.6 + 0.06|*S*_21_|). The lateral dimensions of the PyC film were 7.2 × 3.4 mm^2^, i.e., the film was deposited on the silica substrate that fits precisely the waveguide cross-section; *S*-parameters were measured by subsequent insertion of the specimen into the waveguide.

## Results and discussion

The CVD process parameters and properties of the obtained PyC film are summarized in Table 1.

**Table 1:** Parameters of the CVD process and physical properties of the obtained PyC film

**CH**_**4**_**/H**_**2**_**ratio**	**Press. (mBar)**	**Thickness (nm)**	**Roughness *R***_**a**_**(nm)**	**Optical transmittance at a wavelength of 550 nm**	**Sheet resistance averaged over ten different samples**
75:20	31	25.2 ± 0.8	1.07	37% [8]	200 Ω/sq [8]

**Figure 2:**
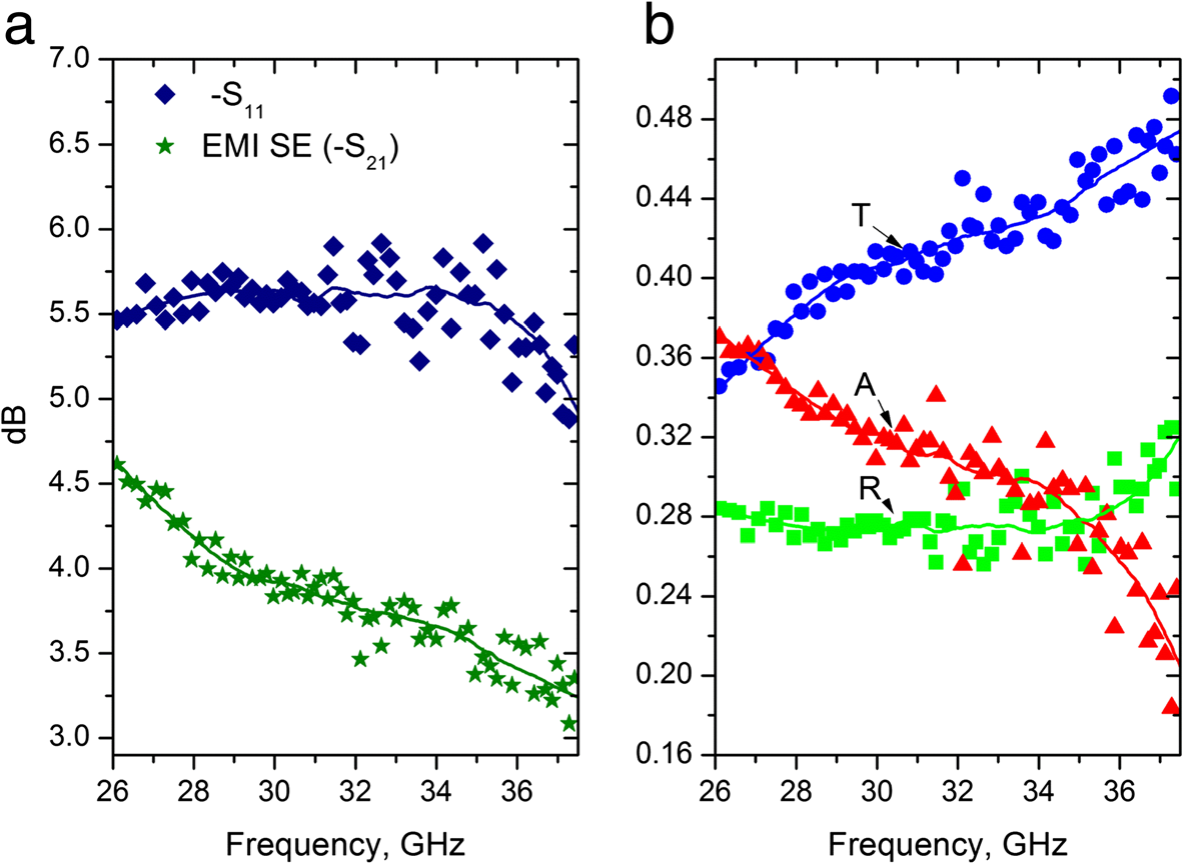
**EM properties of the 25-nm-thick PyC in *K***_**a**_**band.** (**a**) EMI SE and *|S*_11_*|* (**b**) *R* = *|S*_11_*|*^2^, *T* = *|S*_21_*|*^2^, and *A =* 1 *− R − T*. Ratios of transmitted/input (*S*_21_, EMI SE) and reflected/input (*S*_11_) signals measured within 26- to 37-GHz frequency range is presented in (**a**). Reflectivity (*R*), transmitivity (*T*) and absorptivity (*A*) are connected with the measured *S*-parameters as the following: *R* = ***|****S*_11_***|***^2^, *T* = ***|****S*_21_***|***^2^, *A* = 1 − *R* − *T*. Both measured and calculated values of *R*, *T*, and *A* are presented in (**b**).

Ratios of transmitted/input (*S*_21_) and reflected/input (*S*_11_) signals measured within 26- to 37-GHz frequency range (*K*_a_ band) are shown in Figure 2a. Reflectivity *R* = |*S*_11_|^2^, transmittivity *T* = |*S*_21_|^2^, and absorptivity *A =* 1 *− R − T* are presented in Figure 2b. Since the reflectivity and absorptivity of a bare silica substrate are 20% to 25% and 0, respectively, the substrate contribution dominates the reflected signal (approximately 28% of incident power) in Figure 2, while absorption losses are due to the presence of the PyC film. EM absorption of PyC film is found to be as high as 38% to 20% and slightly decrease with the frequency.

It has been shown [7] that absorbance and reflectivity of the free-standing metal film with thickness much less than the skin depth are frequency independent at normal incidence. In our experiment, the frequency dependence of reflectance/absorbance is due to (1) waveguide dispersion and (2) interference in the 0.5-mm-thick silica substrate. The detailed theoretical and numerical analysis of these effects requires taking into account the waveguide modes structure and is beyond the scope of this paper.

Since the film thickness (25 nm) is much smaller than the EM skin depth for conventional metals (a few microns), which is much smaller than the wavelength (1 cm), the PyC film was expected to be transparent to microwaves. However, we found that in the *K*_a_ band, the 25-nm-thick PyC film demonstrates reasonably high absorption losses, which results in the EMI SE as high as 4.75 dB at 26 GHz (see Figure 2a). Thus, the 25-nm-thick PyC film has EMI SE comparable with that of 2.5-μm-thick indium thin oxide film [16]. PyC film microstructure is a key factor affecting its high-frequency conductivity and EMI shielding ability. The intrinsic spatial inhomogeneity of the PyC films results in strong scattering of EM wave that could lead to the ‘anomalous’ absorption.

It is of interest to compare our data with EMI SE of conventional polymers filled with nanocarbon inclusions (carbon nanotubes and carbon onions), which have been recently suggested for conducting and EM interference shielding applications. As it has been shown in [11], the DC conductivity of multiwalled CNT in poly(methyl methacrylate) (PMMA) increases with the carbon mass fraction, showing typical percolation behavior, and EMI SE reaches 5 dB only for 10 wt.% of raw CNT loading at 5 GHz. At room temperature, the high-frequency conductivity of multiwalled CNTs embedded into PMMA in small content (up to 2 wt.%) [17] also turns out to be lower than that of PyC films; only when the concentration reaches 5 wt.% of CNTs in 1-mm-thick PMMA, it provides EMI SE due to absorption at the level of 35%, compatible with that for 25-nm-thick PyC film. Within 1-mm-thick epoxy resin, 0.5 wt.% of single- and multiwalled CNTs gave 2.5 to 2.8 dB of EM attenuation at 30 GHz [18]. Absorbance of carbon onions annealed at high temperatures (1,850 K) embedded in 15 wt.% into 1-mm-thick PMMA/epoxy [19] is the same (approximately 30%) as for 25 nm of PyC film.

## Conclusions

The conductivity of the PyC films at room temperature is comparable with that of the chemically derived graphene flakes and polymers filled with large amount of CNT (5 wt.% and higher). However, in contrast to these carbon-based coatings, the studied PyC film is semi-transparent in visible and infrared ranges. PyC films, being thousands times thinner than the skin depth, provide reasonably high EM attenuation in microwave frequency range due to their high absorptivity. Specifically, the studied 25-nm-thick PyC film absorbs as high as 38% of the incident radiation at 27 GHz. Such an EMI SE is compatible with that of 1-mm-thick coatings containing 1.5 to 5 wt.% of various nanosized carbon forms including graphene nanoplatelets, carbon nanotubes, etc. (see [3] and the references therein). The extremely small thickness and weight of PyC films makes them especially attractive for application in satellite and airplane communication systems. Moreover, PyC films can be deposited on both dielectric and metal substrates of any shape and/or size using conventional and inexpensive CVD technology. Thus, PyC could be used as ultrathin optically semitransparent coatings suitable for *K*_a_ and other microwave frequency bands*.*

## Abbreviations

AFM: atomic force microscopy; CNT: carbon nanotubes; CVD: chemical vapor deposition; EM: electromagnetic; EMI: electromagnetic interference; MW: microwave; PMMA: poly(methyl mathacrylate); PyC: pyrolytic carbon; SE: shielding effectiveness; SEM: scanning electron microscopy.

## Competing interests

The authors declare that they have no competing interests.

## Authors’ contributions

TK and YS produced samples of PyC and studied their physical properties (electrical and optical). AGP and PPK measured EM response properties of PyC films in a microwave range. All authors analyzed the experimental results. PPK, SAM, and YS contributed to the statement of the problem. The manuscript was written primarily by PPK and YS. All authors read and approved the final manuscript.

## Authors’ information

PPK received her M.D. in Theoretical Physics from Belarusian State University in 1991 and Ph.D. degree in Theoretical and High Energy Physics in 1996 from the Institute of Physics, Belarus Academy of Science, Belarus. She is currently a senior researcher at the Research Institute for Nuclear Problems, Belarus State University, Belarus. The general area of her scientific interest is nanoelectromagnetics. She is actively involved in experimental research of electromagnetic response of nanocarbon composite materials in microwave and terahertz ranges. She also contributed to the investigation of electron beam instabilities in CNTs and graphene. She participated in several FP7 projects. AGP received her MS degree in Laser Physics from Belarus State University (BSU), Minsk, Belarus, in 2010, where she is currently working toward the Ph.D. degree. She is also a junior researcher at the Institute for Nuclear Problems, BSU. Her current research interests include dielectric properties of composites with different forms of nanocarbon (single- and multiwalled carbon nanotubes, carbon black, and onion-like carbon) over frequencies ranging from hertz to terahertz. SAM received an MS degree in Physics of Heat and Mass Transfer in 1976, a Ph.D. degree in Theoretical Physics in 1988, both from Belarusian State University, Belarus, and a Doctor of Science degree in Theoretical Physics in 1996 from the Institute of Physics, Belarus National Academy of Science. Since 1992, he has been working as head of the Laboratory of Electrodynamics of Nonhomogeneous Media at the Research Institute for Nuclear Problems, BSU. He also teaches at the BSU Physics Department. He has authored or coauthored more than 150 conference and journal papers. He is a SPIE fellow and is the associate editor of the *Journal of Nanophotonics*. His current research interest is nanoelectromagnetics, which covers the electromagnetic wave theory and electromagnetic processes in quasi-one- and zero-dimensional nanostructures in condensed matter and nanocomposites with the focus on nanocarbon. He participated in a number of international research projects, and is a coordinator of EU FP7 project FP7-226529 BY-NANOERA. TK received his BE degree in Lahti Polytechnics (Finland) in 2005. After finishing his studies in Lahti Polytechnics, he began his studies in the University of Joensuu and graduated with an M.Sc. in Physics in 2009. Since 2010, he has been a Ph.D. student in the University of Eastern Finland working in the field of carbon-based materials. YS received his M.Sc. and Ph.D. in Physics from M. V. Lomonosov Moscow State University (Russia) in 1978 and 1982, respectively. In 1994, he received his DSi degree from the Russian Academy of Science (Moscow). He worked as a senior research fellow at the University of Southampton, UK and University of Tokyo. Since 2001, he has been a professor in Physics at the University of Eastern Finland. He has published about 150 papers in the field of photonics and light-matter interaction.
